# Supplementary material for: Preoperative Chemoradiotherapy for Gastroesophageal Junction Adenocarcinoma Modified by PET/CT: Results of Virtual Planning Study
Source: Medicina (Kaunas). 2021 Dec 6;57(12):1334. doi: 10.3390/medicina57121334 (PMC8705963; doi:10.3390/medicina57121334)
Supplement: Supplementary file 1 [file medicina-57-01334-s001.zip › medicina-1481464-supplementary.pdf]

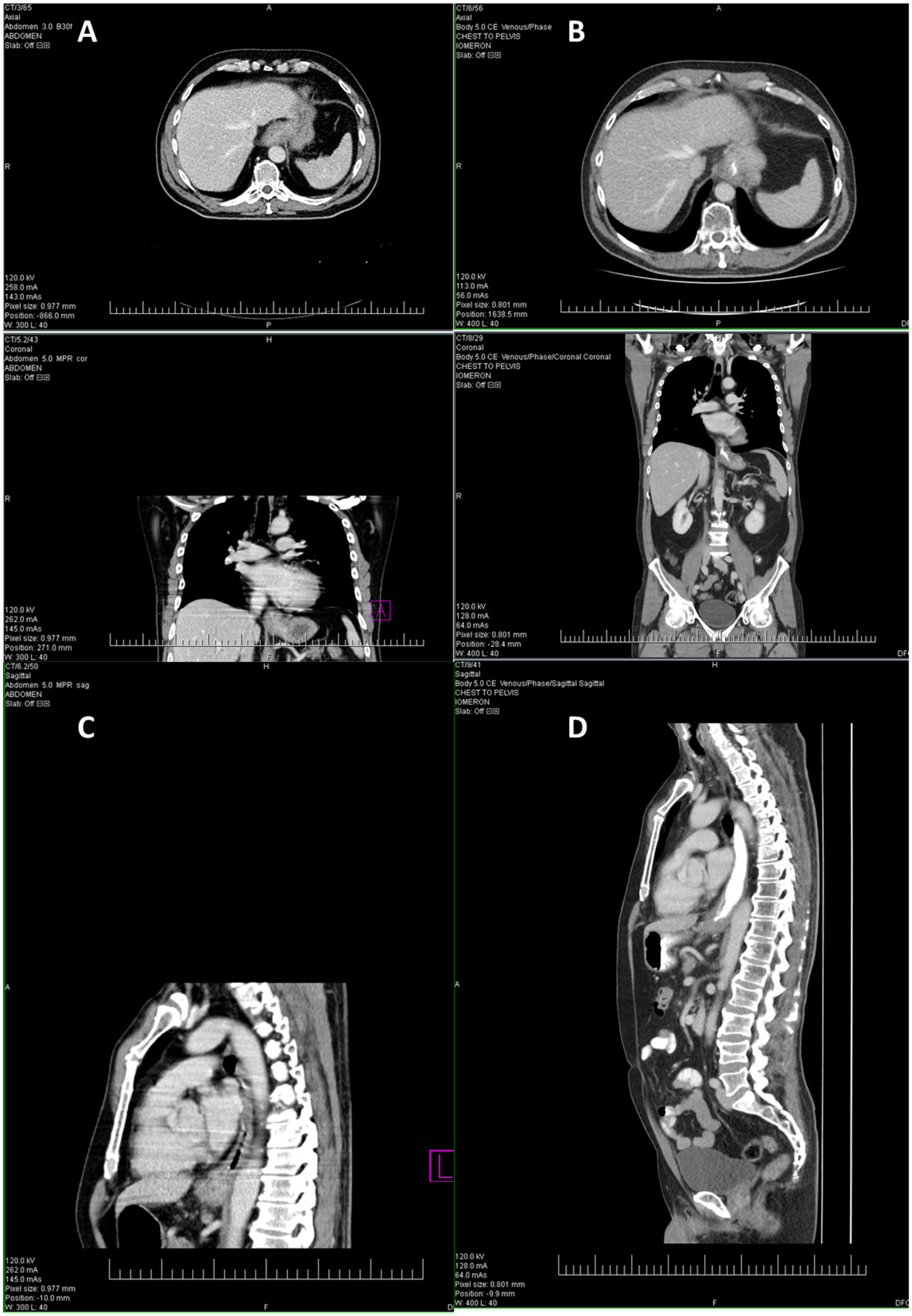

Figure S1. Illustration of (A, C) pre and (B, D)post-radiotherapy CT images for one selected patient. Characteristics: Pre-RT - T4aN1M0, Siewert II, G3 Adenocarcinoma; Post-RT - significant regression, ypT2, ypN3, TRG 3.

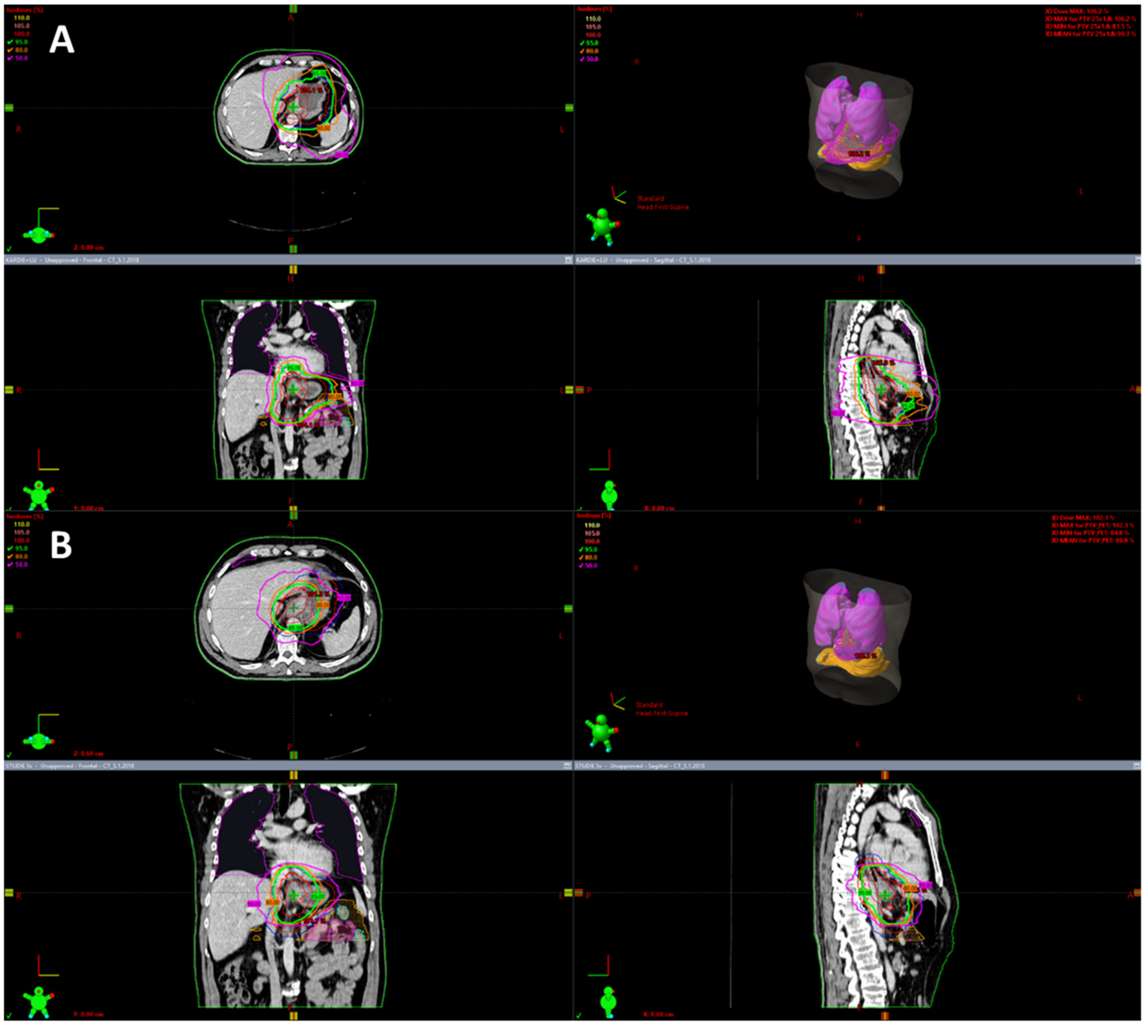

Figure S2. Patient with Siewert II tumor, with suspicious infiltration of a posterior fundal wall, (A) primary plan 45 Gy and (B) additional 9 Gy boost. Green 95% isodose, orange 80% isodose, pink 50% isodose, red GTV and CTV for the boost, blue covered by 95% isodose – PTV boost

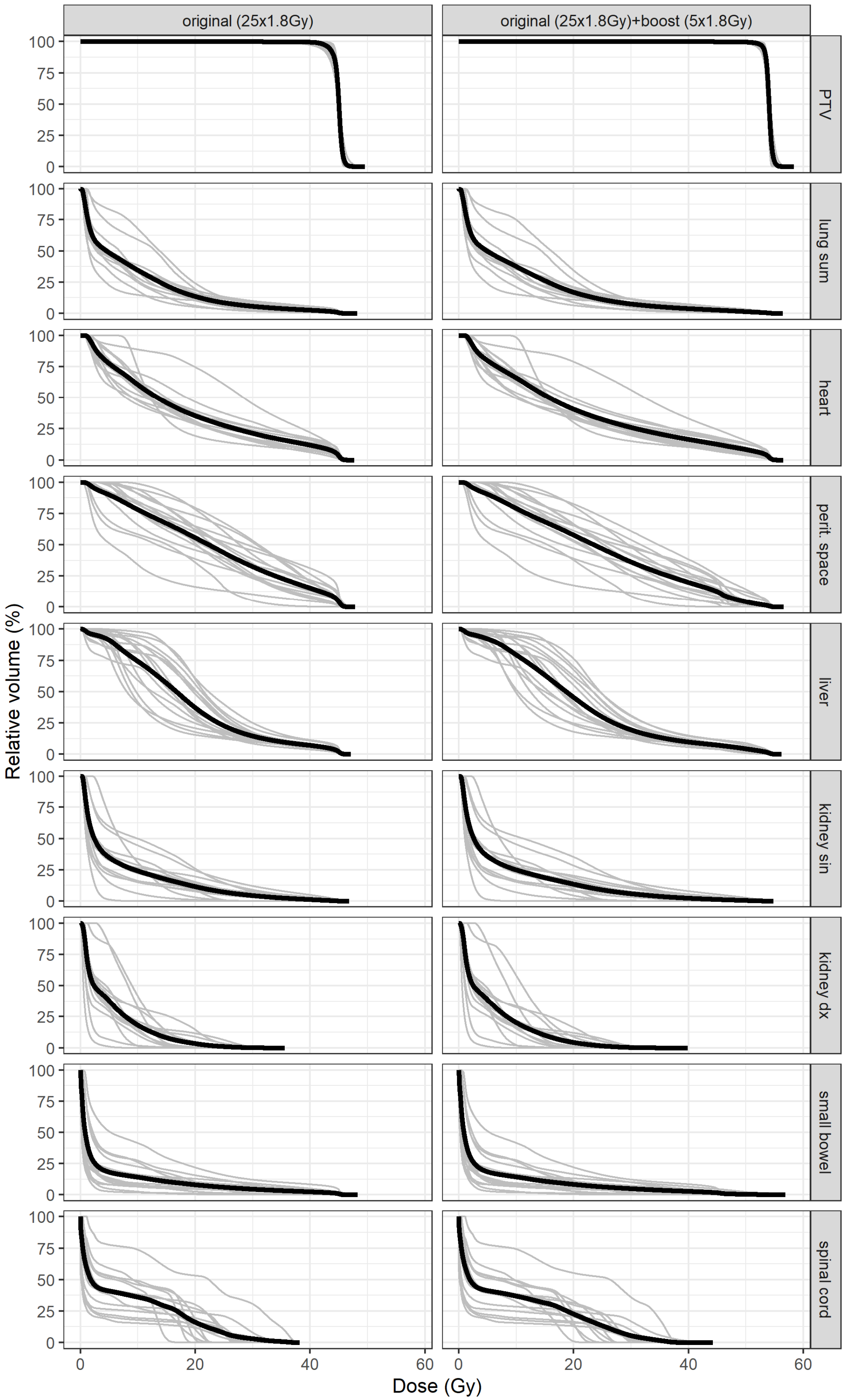

Figure S3. Individual DVHs for each patient (grey) and mean DVH (black) for primary (left) and boost (right) plans.
